# The mirror of illness: identity, embodiment, and clinical care in systemic sclerosis

**DOI:** 10.1007/s10067-026-08236-2

**Published:** 2026-06-23

**Authors:** Neslihan Gokcen

**Affiliations:** Division of Rheumatology, School of Medicine, Sakarya University, Sakarya, Turkey

**Keywords:** Body image, Chronic disease, Identity, Narrative medicine, Patient-centered care, Psychosocial factors, Systemic sclerosis

## Abstract

Systemic sclerosis (SSc) is a complex autoimmune disease characterized by progressive fibrosis, vasculopathy, and multisystem involvement. While clinical assessment appropriately emphasizes measurable outcomes such as organ involvement and survival, these metrics do not fully capture the lived experience of the disease. This perspective examines how SSc reshapes embodiment, identity, and patient experience beyond conventional clinical measures. Drawing on insights from literature and narrative medicine, it explores themes of bodily estrangement, identity disruption, and adaptation in chronic illness. These dimensions have direct implications for patient-physician communication, treatment adherence, and shared decision-making. Integrating narrative awareness into rheumatology practice may enhance clinicians’ ability to recognize psychosocial burden and align care with patient values. A more comprehensive approach to systemic sclerosis, therefore, requires attention not only to measurable disease parameters but also to the meanings patients attach to bodily change and identity over time.

## Systemic sclerosis beyond measurable disease

Systemic sclerosis (SSc) is an uncommon but severe autoimmune connective tissue disease characterized by immune dysregulation, vasculopathy, and progressive fibrosis of the skin and internal organs. Although its population prevalence is relatively low, its clinical and psychosocial impact is disproportionately high [1]. In routine rheumatology practice, disease activity and progression are commonly quantified through the modified Rodnan skin score, pulmonary function testing, imaging, and survival outcomes. These instruments are essential for risk stratification and treatment planning; however, they only partially represent the lived burden of the disease. SSc does not merely alter organ systems. It reshapes embodiment, self-perception, social participation, and the way patients imagine their future [2]. This perspective argues that these dimensions are not peripheral but central to patient-centered care in systemic sclerosis.

SSc progresses along heterogeneous trajectories. Some patients present with primarily cutaneous manifestations; others rapidly develop interstitial lung disease, pulmonary arterial hypertension, gastrointestinal dysmotility, cardiac complications, or renal crisis [1]. This heterogeneity justifies a strong emphasis on objective assessment. Yet the same clinical rigor can unintentionally narrow attention to what is measurable while sidelining what is meaningful to patients.

## The unfamiliar body in chronic illness

For many individuals, the most immediate consequences of SSc emerge in ordinary daily acts: buttoning a shirt with stiffened fingers, maintaining eye contact when facial movement is restricted, or participating in social settings while anticipating comments about visible changes in skin texture and expression. These experiences are not peripheral to disease burden; they are central to it. The loss of manual dexterity may impair professional identity. Facial tightening may reduce not only expressivity but also the patient’s confidence in interpersonal communication. Persistent pain, fatigue, and uncertainty about progression can intensify anxiety and social withdrawal [3, 4]. In this sense, the illness extends beyond pathology and enters biography. As medical humanities scholars have noted, chronic illness does not always lend itself to coherent or redemptive storytelling; some forms of suffering are marked instead by repetition, disruption, and the difficulty of assigning meaning [5].

Although women constitute the majority of patients with SSc, illness perception and adaptation may not be uniform across genders. Emerging evidence suggests that men with SSc may experience distinct challenges related to occupational roles, coping strategies, and support needs, whereas women may experience a greater psychosocial burden related to body image concerns and visible changes in appearance and self-perception. Nevertheless, studies exploring the experiences of men with SSc remain limited, highlighting an important gap in understanding gender-related dimensions of illness experience and adaptation in SSc [6, 7].

Patients with chronic progressive illness often describe a growing distance between the remembered body and the present body. In SSc, this distance can be profound because changes are both functional and visible. The body becomes medically observed and personally unfamiliar at the same time. This duality is clinically important: when patients feel alienated from their own bodies, adherence, hope, and self-efficacy may all be affected.

## Literature as a language for illness

Literature has long served as a lens through which the subjective experience of illness can be explored, revealing dimensions of suffering, identity disruption, and adaptation that remain difficult to capture through biomedical metrics alone.

Literary language offers a precise vocabulary for this phenomenon. In *The Metamorphosis*, Kafka describes the unsettling moment when Gregor Samsa wakes to find himself transformed into an unfamiliar body. Although the transformation in systemic sclerosis is not as sudden, many patients describe a similarly disorienting experience: the gradual feeling that the body they inhabit no longer behaves or appears as it once did. The metaphor is not one of monstrosity but of estrangement, the quiet realization that one’s own body has become unfamiliar.

**Fig. 1:**
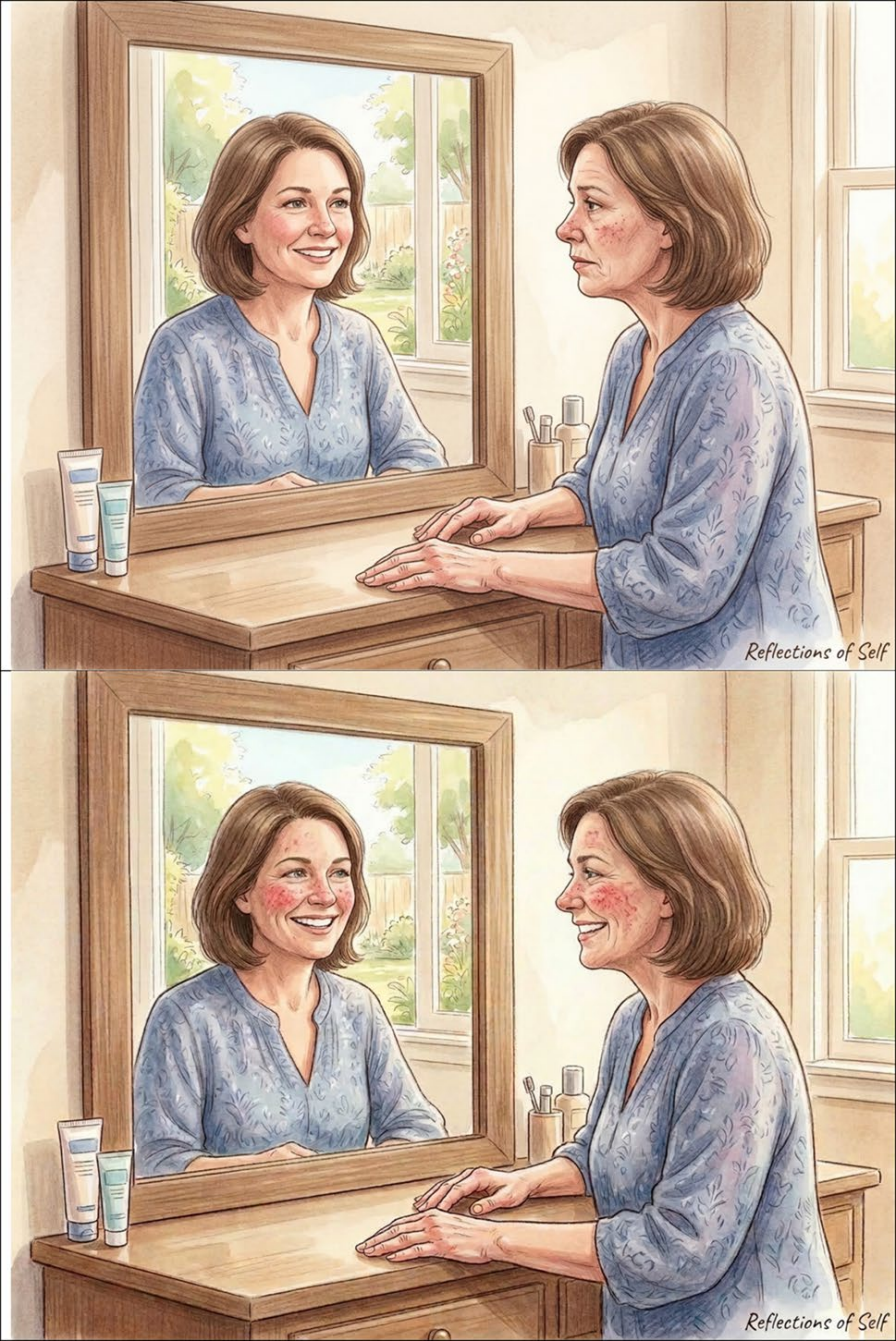
The mirror of illness. Systemic sclerosis may gradually change the contours of the body and the ways in which individuals come to recognize themselves. The illustration symbolizes the distance that can emerge between the remembered body and the present body, as well as the ongoing process of adaptation through which patients seek continuity of identity despite physical transformation

Few objects confront us with change as directly as the mirror. In her poem *Mirror*, Sylvia Plath begins with the stark statement, “I am silver and exact,” portraying the mirror as an uncompromising witness to time and transformation, reflecting without distortion or consolation. For many individuals living with systemic sclerosis, the mirror becomes more than a reflective surface; it becomes a moment of quiet confrontation. The tightening of the skin, the gradual stillness of facial expression, and the subtle reshaping of the hands can transform the familiar landscape of the body into something strangely distant. What medicine records as fibrosis and vascular injury may be experienced by patients as a profound shift in identity. As Plath writes, “In me she has drowned a young girl, and in me an old woman rises toward her day after day.” The poem captures a feeling many patients describe but rarely articulate: the unsettling recognition that the body reflected back from the mirror no longer fully corresponds to the self they remember (Fig. 1).

From a clinical perspective, narrative medicine is not a substitute for evidence-based care; rather, it enriches care by refining clinical listening, deepening interpretive understanding, and supporting ethically grounded, patient-centered decision-making [8]. In SSc, narrative competence can help clinicians identify concerns that are rarely volunteered in standard consultations: shame related to appearance, fear of dependency, grief after occupational loss, strain within intimate relationships, and ambivalence toward escalating immunosuppressive treatment.

Integrating these dimensions into routine care can produce concrete benefits. First, it can improve shared decision-making by linking treatment plans to individual values and roles. Second, it may strengthen trust, especially when disease-modifying options are limited and treatment goals must be negotiated over time. Third, it can guide appropriate referral to multidisciplinary resources, including hand therapy, psychological support, speech and swallow services, and social work. Finally, narrative attention may reduce the risk of therapeutic reductionism, in which a complex person is interpreted solely through a small set of numeric indicators.

For clinicians accustomed to predominantly quantitative approaches, narrative attention may also provide clinically meaningful information that is not readily captured by conventional outcome measures. Understanding how patients experience visible bodily change, altered identity, occupational disruption, or uncertainty may help explain discrepancies between objective findings and perceived disease burden. Such insights may support treatment adherence, improve communication, facilitate shared decision-making, and guide more individualized multidisciplinary care. In this sense, narrative perspectives do not oppose measurement; rather, they complement it by providing contextual understanding of the person behind the disease.

This broader understanding of patient experience also becomes particularly important when considering the psychosocial consequences and adaptive processes that accompany chronic illness. The psychosocial trajectory of SSc is often described in terms of loss, and this framing is justified. Patients may lose comfort, spontaneity, occupational capacity, or prior forms of social confidence. However, clinical encounters should not end with documentation of decline. Meaningful care also requires attention to adaptation, continuity, agency, and the possibility of rediscovering meaning despite change. Some patients develop new forms of self-understanding, re-prioritize relationships, and cultivate resilient identities that are not contingent on full physical restoration.

Derek Walcott offers a useful ethical counterpoint in “Love After Love”: the possibility of meeting oneself again, not by denying change but by integrating it. He writes “you will love again the stranger who was your self.” For many people living with systemic sclerosis, hope does not lie in returning to the body they once knew, but in rediscovering the enduring self that remains beneath the changes illness brings. Hope can also emerge from sustained dignity, relational support, and a therapeutic alliance that acknowledges both the biological severity of the disease and the human depth of the person living with it.

## Meeting the self again

For clinicians, the practical implication is straightforward: assessment should remain biologically rigorous and narratively responsive. Quantitative markers must guide prognosis and treatment, but structured attention to patient narrative should guide communication and care priorities. Brief reflective questions can be incorporated into routine visits without compromising efficiency. Examples include asking which daily activity has become most difficult, what change feels most emotionally significant, and what outcome matters most to the patient in the next 3 to 6 months.

Training programs in rheumatology may also benefit from integrating narrative analysis into education. Exposure to literature and illness narratives can help trainees recognize tacit suffering, tolerate clinical uncertainty, and communicate with greater precision and compassion. Such training does not dilute scientific rigor; it expands clinical intelligence.

SSc challenges medicine at two levels: the technical and the existential. At the technical level, clinicians must manage a complex multisystem disease using evolving evidence and multidisciplinary coordination. At the existential level, they must care for persons whose identities, relationships, and futures are being renegotiated under the pressure of chronic illness. This perspective highlights that literature and narrative approaches do not replace clinical data, but rather illuminate dimensions of illness that conventional measures cannot fully capture. Recognizing these dimensions may improve patient-physician communication, strengthen shared decision-making, and support more person-centered care. A mature model of systemic sclerosis care, therefore, requires both measurement and meaning: the careful interpretation of organs and the equally careful listening to the persons who live within them.

## Data Availability

Not applicable.
